# Structure Analyses of Fe-based Metallic Glasses by Electron Diffraction

**DOI:** 10.3390/ma3125263

**Published:** 2010-12-13

**Authors:** Akihiko Hirata, Yoshihiko Hirotsu

**Affiliations:** 1 WPI Advanced Institute for Materials Research, Tohoku University, Sendai 980-8577, Japan; 2 Osaka University Groningen Centre for Education and Research, Zernikelaan 6, 9747 AA, Groningen, The Netherlands; E-Mail: hirotsu@osaka-u-groningen.org

**Keywords:** metallic glass, local atomic structure, nanobeam electron diffraction, pair distribution function analysis

## Abstract

Nanoscale structural information of amorphous structures has become obtainable by using nanobeam electron diffraction in combination with high resolution imaging. In addition, accurate radial distribution function analysis using energy filter has also become available to know averaged amorphous structures. In this paper, we introduce some applications of these techniques, especially to several Fe-based metallic glasses. On the basis of these results, we discuss a relationship between the glass structure and the glass stability in Fe-based metallic glasses

## Introduction

Recently, bulk metallic glasses [1] of metal-metal and metal-metalloid multi-component systems with clear glass transitions have been attracting much interest with their excellent mechanical and physical properties, and in these glasses local atomic arrangements are considered to play a crucial role for the stabilization of glass-phases. A number of X-ray and neutron diffraction structure analyses have been performed for both the metal-metal and metal-metalloid metallic glasses [2,3]. Atomic short range order (SRO) with respect to the nearest neighbor structures has been elucidated through radial distribution function (RDF) analyses for the glass states. In addition to this, crystallization processes, especially in nanoscale, also become important for understanding changes of mechanical and physical properties [4,5]. Even for specimens with nanocrystallized structures, however, such average structural analyses have been performed by focusing on changes of the SRO structures [6]. To understand inhomogeneity of nanoscale structures, including medium range order (MRO) structures or very small nanocrystals, we should carry out atomistic-scale local structural analyses by means of advanced transmission electron microscopy (TEM), taking the averaged structure information from the RDF analysis into consideration.

One of the advantages of using modern TEM is to obtain structural information from local regions as small as 1 nm by using nanobeam electron diffraction (NBED) technique. This technique enables us even to take crystalline symmetric diffraction patterns from 1 nm-sized atomic ordered regions in as‑formed glass structures, which directly proves a presence of extended MRO structures [7,8]. Moreover, average structural information can also be obtained precisely by selected area diffraction (SAED) patterns from wide areas (0.1~1 μm) of the specimens. The RDF analysis from SAED intensity and reverse Monte Carlo (RMC) simulation enables us to construct plausible structural models as average information [9]. Owing to advanced technologies such as energy filtering and digital data processing with imaging-plates (IP), precise intensity analyses become available even by electron diffraction. A simultaneous observation in both real and reciprocal spaces for the same region of interest is a great advantage in the structure analyses using TEM. In other words, both of the imaging and diffraction techniques are concurrently available for identical regions in the specimens. Therefore, we can always monitor a degree of inhomogeneity of the microstructure in real space which corresponds to the RDF profile of averaged structure. Using these techniques, we have revealed both local and average atomic structures in the glass and nanocrystallized specimens. In this paper, we first briefly explain the analytical techniques developed by our group, and then introduce some examples of the analyses especially for Fe-based metallic glasses.

## 2. Experimental Procedure

### 2.1. Structural Observation

For TEM and electron diffraction studies, the ribbon and bulk metallic glass specimens were thinned by using both electrolytic polishing (with acetic and perchloric acids) and Ar-ion thinning techniques. Note that the results obtained from these different thinning techniques basically show the same image and diffraction features. In the ion-thinning, a specimen stage capable of low glancing‑angle ion-thinning with a low voltage of 200 V was used in the final thinning. The SAED patterns were recorded on IP in a LEO-922D TEM equipped with an omega-type energy-filter. The SAED patterns were recorded on IP and read using IP readers. NBED patterns were obtained by 300 kV TEM (JEM‑3000F). A full width at half maximum of the nano-probe in NBED was about 1 nm, which was achieved by using a field emission gun and a small condenser aperture with a size of 10 μm. The amount of electron dose for the nano-probe in NBED was 2.0 × 10^19^ e/cm^2^ (measured by a Faraday gauge), almost 10 times smaller than that of the conventional high-resolution electron microscope imaging. The NBED patterns were recorded using a TV-rate camera by scanning the nano‑probe on the specimen continuously with a scanning speed of about 10 nm /s.

### 2.2. PDF Analysis from Electron Diffraction Intensity

The diffraction intensity was recorded on IPs. The diffraction intensities was then read as the observed intensity *I_obs_*(*Q*) for the scattering vector *Q* using the relationship, *Q* = 4π*sinθ*/λ=(2πR/*L*λ)(1-3*R*^2^/8*L*^2^), where θ is the half scattering angle and λ the electron wave length. From the observed elastic intensity *I_obs_ (Q)*, the interference function *i(Q)* can be expressed as in Equation (1) using the usual formula of *i(Q)* [2], where *BG*(*Q*) corresponds to the background intensity proportional to $N\langle f^{2}(Q)\rangle$.

(1) $$i(Q)=\frac{I_{obs}(Q)-\mathrm{BG}(Q)}{\mathrm{BG}(Q)\;{\langle f(Q)\rangle }^{2}/\langle f^{2}(Q)\rangle }$$

**Figure 1:**
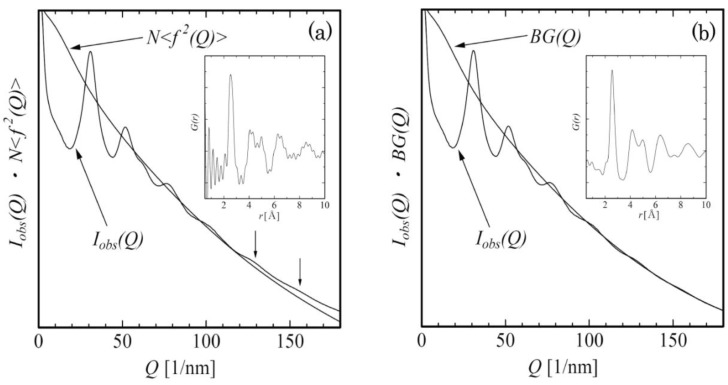
(**a**) Background profile proportional to atomic scattering factor and (**b**) slightly modified background profile, together with the electron diffraction intensity obtained from an Fe_80_B_20_ metallic glass. In each inset, reduced pair distribution functions, which are derived by Fourier transform of background-subtracted intensity profiles (reduced interference functions), are shown.

Here *Q* is the scattering vector and *N* means atom number. The square-mean and mean-square atomic scattering factors are expressed as $\langle f^{2}(Q)\rangle =\sum N_{j}f_{j}^{2}(Q)/N$ and ${\langle f\rangle }^{2}=(\sum N_{j}f_{j}{\left(Q)\right)}^{2}/N^{2}$, respectively. The background intensity profiles obtained from the thin specimen areas in this study varied basically along the $N\langle f^{2}(Q)\rangle$ curve, although the curve slightly deviates from the *I_obs_(Q)* at high-*Q* region. We had already checked that the slight deviation is mainly ascribed to a residual inelastic scattering and multiple scattering. This can be seen in Figure 1 (a) for the *I_obs_*(*Q*) profile together with $N\langle f^{2}(Q)\rangle$ for the amorphous Fe_80_B_20_ specimen. At the high-Q region, we can see a slight oscillation in the *I_obs_*(*Q*) profile indicated by arrows. To precisely read the information of the slight oscillation, a slight modification of the background intensity is necessary in the following way. The *i(Q)* can be transformed to the total reduced pair distribution function as

(2) $$G(r)=\frac{2}{\pi }\int_{0}^{\infty }Qi(Q)\sin(Qr)dQ=4\pi r[\rho (r)-\rho_{0}]$$

where *ρ(r)* is the atomic density and *ρ_0_* the average atomic density which can be obtained from the specimen physical density. The *G*(*r*) profile, obtained from the Fourier transform of *Qi_obs_(Q)* using Equations (1) and (2), gave a ripple near *G*(*r*) with *r~*0. This is because the background intensity cannot be drawn correctly and a redrawing is necessary generally in such a case. We then modified the background to give *G*(*r*) with smaller ripples near *r~*0, and this procedure is repeated. The final background intensity *BG(Q)* is drawn in Figure 1 (b), together with a corresponding *G(r)* profile where the ripple near *r*~0 was sufficiently reduced [10].

### 2.3. Reverse Monte-Carlo Simulation

For the RMC simulation of Fe-based metallic glasses, we first prepared random (or relaxed) atomic arrangements with 5,000 atoms in cubic cells consistent with alloy physical densities for the initial input. In the RMC procedure [9], a fitting of a calculated total interference function *i_cal_*(Q) (or PDF*_cal_*) obtained from a structural model is performed to the experimentally obtained *i_exp_*(Q) (or PDF*_exp_*). In the fitting procedure, *i_cal_*(Q) (or PDF*_cal_*) is compared with *i_exp_*(Q) (or PDF*_exp_*) for every event of atomic positional change, and the atomic move is accepted to be preferable if the move resulted in a decrease of the mean square deviation, χ^2^, between *i_exp_*(Q) and *i_cal_*(Q). Even if χ^2^ increases, the atomic movement was accepted with a probability of exp (-(χ_new_^2^ - χ_old_^2^) / 2). Otherwise the movement was rejected. Here, χ^2^ was defined as χ^2^ = ∑ (*i_exp_*(*Q*) - *i_cal_*(*Q*))^2^/σ (*Q*), where σ (Q) is the experimental error.

## 3. Results and Discussion

We have been systematically examining local atomic structures on Fe-based metallic glasses with special significance as magnetic materials, by using the above-mentioned advanced TEM techniques. As a first step, we researched the simple Fe-B binary system, for which there are fruitful and reliable data examined by neutron and X-ray diffraction methods. In this study, Fe-B alloys with three different compositions were used to examine a compositional dependence of the local atomic structure. Although bcc-like clusters extending to about 1 nm were frequently observed in high‑resolution electron microscope (HREM) images [11], it was difficult to find any difference in the HREM images. From electron diffraction intensity analyses, however, we found detectable structural differences in these alloys. The obtained results are described below.

**Figure 2:**
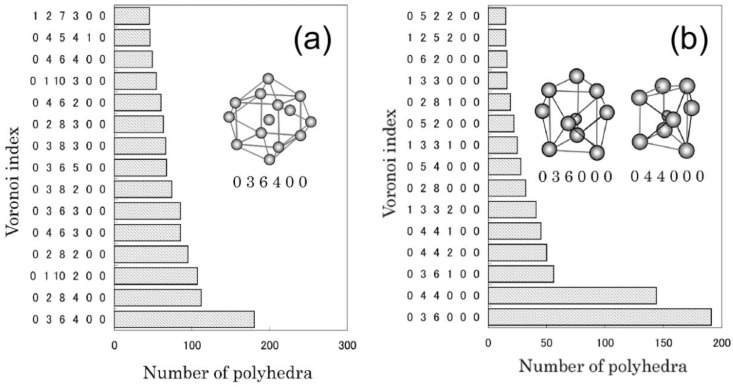
Voronoi polyhedral analyses for the reverse Monte Carlo (RMC) structure model which reproduces the interference function *i(Q)* obtained from Fe_80_B_20_. Voronoi polyhedra with central (**a**) Fe and (**b**) B atoms found in the final structural model are shown.

Plausible structure models for Fe_86_B_14_, Fe_83_B_17_, Fe_80_B_20_ alloys were constructed [10] using the above mentioned RMC simulation technique. The coordination numbers and bond lengths are well consistent with those reported based on the neutron data [12]. To understand the local atomic environments, a Voronoi polyhedral analysis [13] was performed for the obtained structural models. Figure 2 shows the analyzed results obtained from the model for Fe_80_B_20_. The bcc-Fe like polyhedra are frequently found around central Fe atoms, whereas the prism-type polyhedra are frequently formed around central B atoms. The trigonal prism, which is a structural unit of an Fe_3_B compound, increases with the increment of B content, as well as Archimedean prism. In contrast, the bcc-Fe like polyhedra increase with the decrease of B content. It is interesting to note that the change of ratio of the bcc-Fe and prism type local structures with the B content basically corresponds to the change of moler ratio of bcc-Fe and Fe_3_B compound with the B composition in the equilibrium Fe-B phase diagram. In each alloy, moreover, icosahedron (coordination number is 12) and polyhedra with large coordination numbers also exist.

**Figure 3:**
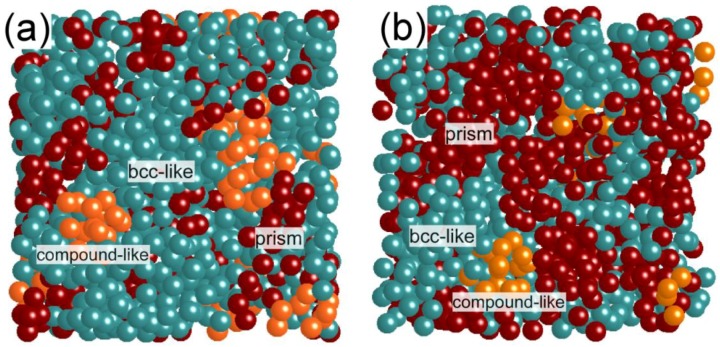
Structure models of (**a**) Fe_84_Nb_7_B_9_ and (**b**) Fe_70_Nb_10_B_20_ metallic glasses obtained from the RMC simulation. A fraction of the bcc-Fe clusters in Fe_70_Nb_10_B_20_ is smaller than that in Fe_84_Nb_7_B_9_.

It was confirmed that the precise electron diffraction intensity analyses can be performed for the Fe‑B binary system as above. We then moved our subjects to ternary Fe-B-Nb alloys in order to clarify a difference in local atomic structures of metallic glasses with various glass stabilities [14]. We focused especially on ternary Fe_84_Nb_7_B_9_ and Fe_70_Nb_10_B_20_ glass structures [15,16], with a structural reference of Fe_80_B_20_. The Fe_70_Nb_10_B_20_ glass has a high glass forming ability, showing a large supercooled liquid region. Structural models for the Fe_84_Nb_7_B_9_ and Fe_70_Nb_10_B_20_ glasses were also derived using the RMC simulation, and a reliability of the models was confirmed by checking the consistency of average coordination numbers between the simulation and experiments. Even for ternary alloys, experimental coordination numbers and bond lengths can be obtained by using an X-ray anomalous dispersion method or a neutron diffraction method [17]. We actually obtained the RMC simulated model for ternary Fe_70_Nb_10_B_20_, which is well consistent with the results from an X-ray anomalous dispersion method [17]. Voronoi polyhedral analyses for these glasses revealed that local atomic structures have “nanoscale-phase-separated” features which basically consist of bcc-Fe like clusters, trigonal prisms, and compound-like polyhedra. Note that the fraction for each cluster strongly depends on the alloy composition: for example, the fraction of bcc-Fe clusters in Fe_70_Nb_10_B_20_ is much smaller than that in the other glasses. Figure 3 visually shows the atomic structures for Fe_84_Nb_7_B_9_ and Fe_70_Nb_10_B_20_ obtained from the RMC simulation. It can be seen that a fraction of the bcc-Fe like clusters in Fe_70_Nb_10_B_20_ has a small proportion compared to Fe_84_Nb_7_B_9_. Moreover, we can see trigonal prisms and compound-like polyhedra between aggregates of bcc-Fe like clusters, indicating a formation of the structures characterized by “nanoscale phase separation” [8]. This kind of structural fluctuation probably gives a strong effect on the crystallization processes of these glasses [18].

In addition to the local structural analyses, we have been trying to understand the glass stability from a viewpoint of the crystallization processes. We also examined multicomponent metallic glasses, such as Fe_72_Si_4_B_20_Nb_4_ and Fe_36_Co_36_Si_4_B_20_Nb_4_, with large bulk glass forming abilities, much larger than the above binary and ternary glasses. As a result, we found a distinct difference in crystallization processes between glasses with (multicomponent) and without (binary and ternary) bulk glass forming abilities, as follows. The complex intermetallic compound phases are directly formed from the glass states in the bulk metallic glasses, whereas the simple bcc-Fe phase is initially formed prior to the compound formation in the metallic glasses without bulk forming abilities. An example of the crystallization process in one of the multicomponent bulk metallic glasses, will be shown below.

**Figure 4:**
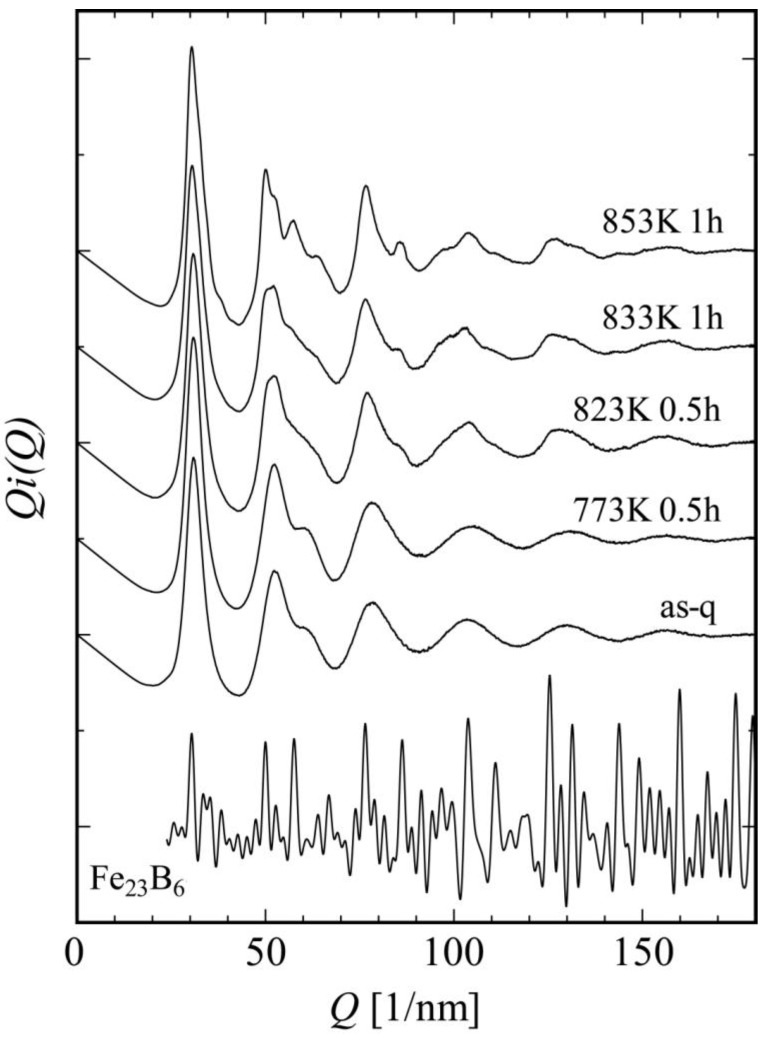
Change of reduced interference functions obtained from Fe_36_Co_36_Nb_4_Si_4_B_20_ on heating. The glass state directly transforms into a metastable Fe_23_B_6_ crystal.

**Figure 5:**
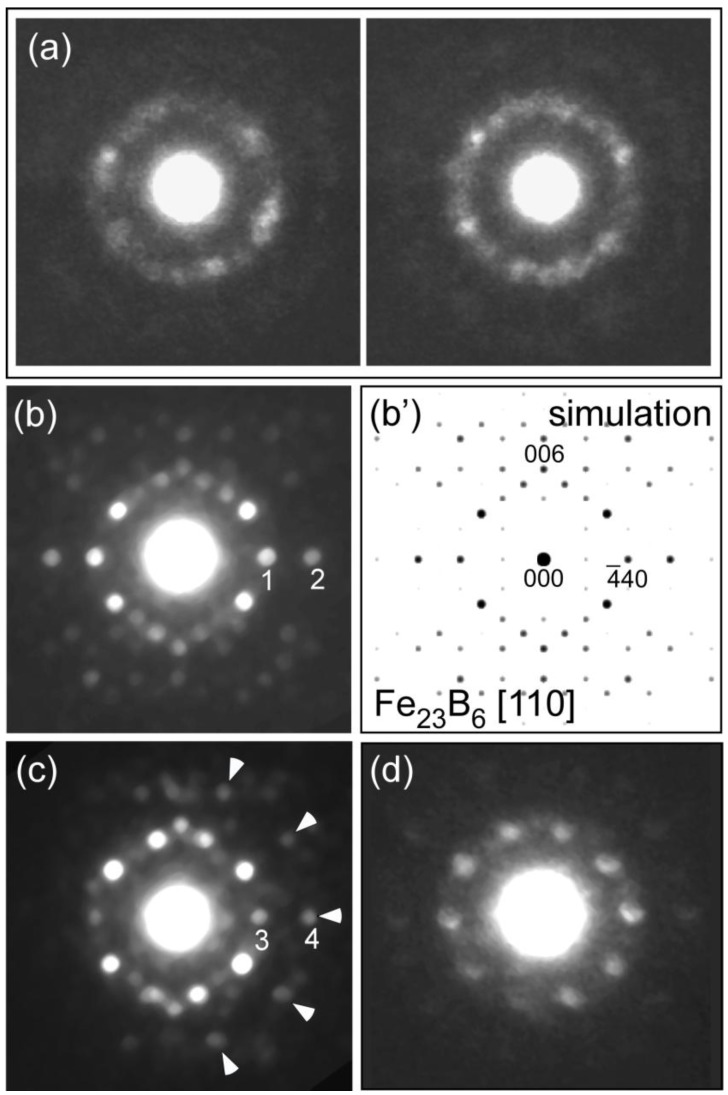
(**a**) NBED patterns obtained from as-quenched Fe_36_Co_36_Nb_4_Si_4_B_20_ specimen. NBED patterns obtained from Fe_36_Co_36_Nb_4_Si_4_B_20_ specimens annealed at 873K for 1h are also shown in (b)-(d). (**b**) [110] pattern from a perfect Fe_23_B_6_ structure, (**c**) Fe_23_B_6_–like pseudo-tenfold pattern, and (**d**) almost tenfold pattern. The simulated [110] pattern of Fe_23_B_6_ is also shown in (**b’**).

We observed microstructures of as-quenched and annealed Fe_36_Co_36_Si_4_B_20_Nb_4_ metallic glass [], which can form a bulk rod glass with a diameter as large as 4 mm [21]. In the as-quenched specimen, HREM images indicate a homogeneous glass structure where extended MRO structures cannot be seen clearly. Figure 4 shows a change in interference function *i(Q)* on the annealing process of Fe_36_Co_36_Si_4_B_20_Nb_4_. In this alloy system, it was found that the glass structure directly transforms to Fe_23_B_6_ (cF116 Cr_23_C_6_-type) nanocrystals without a formation of bcc-Fe. To understand the nanoscale structural changes, we next took a lot of NBED patterns from a broad range of areas in each specimen. Diffraction spots with strong intensities are frequently observed just on the first- and second-halo-ring positions in NBED patterns, indicating a development of the MRO structures even in the as-quenched state (Figure 5 (a)). Contrary to the metallic glasses with low glass forming abilities, crystal-like (including bcc-Fe) diffraction patterns cannot be obtained even by a careful inspection of a lot of NBED patterns. It seems that there are locally extended MRO structures to form NBED spots arranged on the halo-ring positions without any specific atomic structure with high symmetry detectable by NBED. Figure 5 (b) shows an [110] NBED pattern from a metastable Fe_23_B_6_-type structure in the specimen annealed at 873 K, together with a corresponding calculated pattern in (b’). A NBED pattern, slightly deformed from the perfect Fe_23_B_6_ one, is shown in Figure 5 (c). In the pattern, diffraction spots indicated by arrows form a pattern with pseudo-tenfold symmetry in which a proportion of Q values between spots 3 and 4 is about 1.62. This value is very close to the golden ratio (1.618), which characterizes quasicrystals, and is far from a normal value of 1.5 (proportion between spots 1 and 2) found in the perfect Fe_23_B_6_ structure. This means that, even in the Fe-based metallic glass, the quasicrystal-like structure can be found, as well as in the Zr-based metallic glasses. Moreover, we found the quasicrystal-like structure also in an Fe-Cr-Mo-C-B-Tm metallic glass with an extremely high glass forming ability [22,23]. It is therefore concluded that the glass nature to form a quasicrystal structure in crystallization stage is closely related to the glass formation as was previously discussed in the Zr-based systems [24].

**Figure 6:**
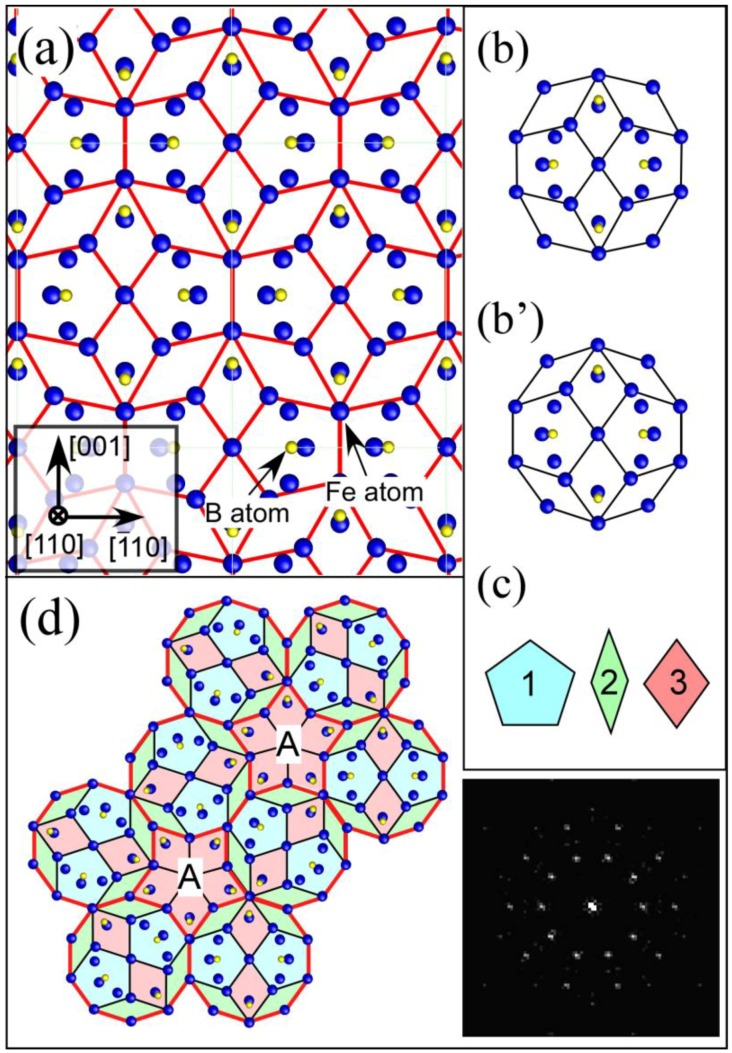
(**a**) [110] projection of an Fe_23_B_6_ structure; (**b**) deformed decagonal structural unit found in (a); (**c**) decagonal structural unit obtained by transforming the unit of (b), three types of structure tiles as components of the decagonal unit of (c); and (**e**) structure model reproducing the tenfold diffraction pattern shown in Figure 5 (**d**). For the structure model of (e), we first arrange several decagonal structure units (c), and then rotated them by 0, 72, 144, 216, 288 degrees independently. These tiles were subsequently arranged to fill the space by sharing their edges. Though the decagonal structure unit cannot fill the space denoted by A, the space can be filled by using the tile denoted by "3". As a result, three types of tiles shown in (c) can form the structure which reproduces the tenfold diffraction pattern. In the inset, Fourier transform pattern obtained from the structure model is shown. The pattern is well consistent with the experimental diffraction pattern.

Interestingly, the [110] projection of the Fe_23_B_6_ structure (Figure 6 (a)) can be divided into three types of characteristic tiles shown in Figure 6 (c). These tiles are able to form a quasicrystal-like structure exhibiting a pseudo-tenfold symmetry as shown in Figure 6 (d). During the formation process of the Fe_23_B_6_ structure, quasicrystal-like clusters, which have strong correlation with the stable glass structure, are probably stabilized as an intermediate state. We need to focus on the fact that the formation of the intermediate quasicrystal structures in Fe-based systems is very similar to that in the Zr-based systems.

## 4. Conclusions

By using advanced TEM techniques, we performed both average and local structural analyses for several Fe-based metallic glasses. For simple binary and ternary metallic glasses, the electron diffraction intensity analyses (average structural analyses) revealed clear compositional dependence of fractions for each characteristic atomic polyhedron (prism, bcc-like, icosahedron, compound-like, *etc.*) with the help of the RMC simulation. Nanobeam diffraction analyses (local structural analyses) clearly revealed complicated local structural changes in early stages of crystallization in the multicomponent metallic glasses. Owing to this technique, we found nanoscale quasicrystal-like structures in the metal‑metalloid type Fe-based metallic glasses for the first time. We conclude that both of the average and local structural analyses using advanced TEM are very useful to understand the local structural features in metallic glasses. A more precise local NBD structure analysis with a smaller size of nano‑probe (less than 1 nm in probe-diameter) will be necessary to investigate detailed features of MRO structures, in conjunction with the higher resolution HREM imaging, using Cs-corrected TEM, to directly visualize the local structures.

## References

[1] Inoue A. (2000). Stabilization of metallic supercooled liquid and bulk amorphous alloys. Acta Mater..

[2] Elliott S.R. (1990). Physics of amorphous materials.

[3] Waseda Y. (1980). The Structure of Non-Crystalline Materials, Liquids and Amorphous Solids.

[4] Makino A., Inoue A., Masumoto T. (1995). Nanocrystalline Soft-Magnetic Fe-M-B (M = Zr, Hf, Nb) Alloys Produced by Crystallization of Amorphous Phase (Overview). Mater. Trans. Jim.

[5] Inoue A., Wang X.M. (2000). Bulk amorphous FC20 (Fe-C-Si) alloys with small amounts of B and their crystallized structure and mechanical properties. Acta Mater..

[6] Schumacher H., Herr U., Oelgeschlaeger D., Traverse A., Samwer K. (1997). Structural changes of the metallic glass Zr_65_A_l7.5_Cu_27.5_ during glass transition and in the undercooled liquid region. J. Appl. Phys..

[7] Hirotsu Y., Ohkubo T., Matsushita M. (1998). Study of amorphous alloy structures with medium range atomic ordering. Microsc. Res. Tech..

[8] Ohkubo T., Hirotsu Y. (2003). Electron diffraction and high-resolution electron microscopy study of an amorphous Pd_82_Si_18_ alloy with nanoscale phase separation. Phys. Rev. B.

[9] McGreevy R.L. (2003). Reverse Monte Carlo modeling. J. Phys..

[10] Hirata A., Hirotsu Y., Matsubara E., Ohkubo T., Hono K. (2006). Mechanism of nanocrystalline microstructure formation in amorphous Fe-Nb-B alloys. Phys. Rev. B.

[11] Hirotsu Y, Akada R. (1984). High resolution electron microscopic observation of microcrystalline domains in an amorphous Fe_84_B_16_ alloy. Jap. J. Appl. Phys..

[12] Nold E., Lamparter P., Olbrich H., Rainerharbach G., Steeb S. (1981). Determination of the Partial Structure Factors of the Metallic-Glass Fe_80_B_20_. Z. Naturforsch. Sec. A.

[13] Finney J.L. (1979). Procedure for the Construction of Voronoi Polyhedra. J. Comp. Phys..

[14] Matsubara E., Sato S., Imafuku M., Nakamura T., Koshiba H., Inoue A., Waseda Y. (2000). Anomalous X-ray scattering study of amorphous Fe_70_M_10_B_20_ (M = Zr, Nb, and Cr) alloys. Mater. Trans. Jim.

[15] Hirata A., Hirotsu Y., Matsubara E. (2005). Local atomic structures of amorphous Fe_80_B_20_ and Fe70Nb10B20 alloys studied by electron diffraction. Mater. Trans..

[16] Hirata A., Hirotsu Y., Ohkubo T., Matsubara E., Makino A. (2006). Local structure studies of Fe-Nb-B metallic glasses using electron diffraction. J. Microscopy (Oxford).

[17] Imafuku M., Sato S., Matsubara E., Inoue A. (2002). Structural study of Fe_90-x_Nb_10_B_x_ (x = 10, 20 and 30) glassy alloys. J. Non-Cryst. Solids.

[18] Hirata A., Hirotsu Y., Matsubara E., Ohkubo T., Hono K. (2006). Mechanism of nanocrystalline microstructure formation in amorphous Fe-Nb-B alloys. Phys. Rev. B.

[19] Hirata A., Hirotsu Y., Amiya K., Nishiyama N., Inoue A. (2008). Nanocrystallization of complex Fe_23_B_6_-type structure in glassy Fe-Co-B-Si-Nb alloy. Intermetallics.

[20] Hirata A., Hirotsu Y., Amiya K., Nishiyama N., Inoue A. (2009). Fe_23_B_6_-type quasicrystal-like structures without icosahedral atomic arrangement in an Fe-based metallic glass. Phys. Rev. B.

[21] Amiya K., Urata A., Nishiyama N., Inoue A. (2007). Magnetic properties of Co-Fe-B-Si-Nb bulk glassy alloy with zero magnetostriction. J. Appl. Phys..

[22] Hirata A., Hirotsu Y., Amiya K., Inoue A. (2008). Crystallization process and glass stability of an Fe_48_Cr_15_Mo_14_C_15_B_6_Tm_2_ bulk metallic glass. Phys. Rev. B.

[23] Hirata A., Hirotsu Y., Amiya K., Inoue A. (2009). Nanoscale metastable state exhibiting pseudotenfold diffraction pattern in Fe-based bulk metallic glass. Phys. Rev. B.

[24] Dong C., Wang Q., Qiang J.B., Wang Y.M., Jiang N., Han G., Li Y.H., Wu J., Xia J.H. (2007). From clusters to phase diagrams: Composition rules of quasicrystals and bulk metallic glasses. J. Phys. D.

